# Chromosome Mapping of 5S Ribosomal Genes in Indo-Pacific and Atlantic Muraenidae: Comparative Analysis by Dual Colour Fluorescence In Situ Hybridisation

**DOI:** 10.3390/genes11111319

**Published:** 2020-11-06

**Authors:** Elisabetta Coluccia, Federica Deidda, Cinzia Lobina, Riccardo Melis, Cristina Porcu, Blondine Agus, Susanna Salvadori

**Affiliations:** Department of Life and Environmental Sciences, University of Cagliari, via Tommaso Fiorelli 1, 09126 Cagliari, Italy; federica.deidda@unica.it (F.D.); cinzia.lob@email.it (C.L.); riccardo.melis@unica.it (R.M.); cporcu@unica.it (C.P.); blondine.agus@unica.it (B.A.); salvador@unica.it (S.S.)

**Keywords:** comparative cytogenetics, FISH, *Gymnothorax*, *Muraena*, Muraenidae, ribosomal genes

## Abstract

The Muraenidae is one of the largest and most complex anguilliform families. Despite their abundance and important ecological roles, morays are little studied, especially cytogenetically, and both their phylogenetic relationships and the taxonomy of their genera are controversial. With the aim of extending the karyology of this fish group, the chromosomal mapping of the 5S ribosomal gene family was performed on seven species belonging to the genera *Muraena* and *Gymnothorax* from both the Atlantic and Pacific oceans. Fluorescence in situ hybridisation (FISH) experiments were realized using species-specific 5S rDNA probes; in addition, two-colour FISH was performed to investigate the possible association with the 45S ribosomal gene family. Multiple 5S rDNA clusters, located either in species-specific or in possibly homoeologous chromosomes, were found. Either a syntenic or different chromosomal location of the two ribosomal genes was detected. Our results revealed variability in the number and location of 5S rDNA clusters and confirmed a substantial conservation of the number and location of the 45S rDNA.

## 1. Introduction

Within the teleosts, the Elopomorpha—tarpons, eels, and relatives—is considered to be one of the major extant taxa, besides the Osteoglossomorpha and Clupeocephala. The Elopomorpha is the sister group to the rest of the teleosts [1]. This group includes about 1000 species, with Anguilliformes being the most diverse order, containing over 90% of the species [2]. Monophyly of the group is currently accepted based on both molecular and morphological evidence; their most remarkable synapomorphy is the leptocephalus larva [1,3]. The phylogenetic relationships among the approximately 1000 species, and their taxonomy, remain largely unresolved [1,4,5,6]. Within the Anguilliformes order, four monophyletic groups, the Protanguilloidei, Muraenoidei, Anguilloidei, and Congroidei, have been recognized [1].

The Muraenidae is one of the largest and most complex anguilliform families, with 219 extant species [2]. Moray eels are very common in coastal tropical and temperate seas around the world, and are key mesopredators in coral reefs and rocky environments, feeding on a wide diversity of vertebrates (fish) and invertebrates (crustaceans and cephalopods) [7]. This family comprises 16 genera, separated into two subfamilies: Muraeninae, with 164 species, and Uropterygiinae, with 36 species. The genus *Gymnothorax* is the most species rich, with 126 species [8,9]. Monophyly of this ancient family, whose appearance dates between 54 and 34 million years ago, and of the two subfamilies, is well supported, unlike other families, such as the Congridae [4,5,10].

Despite their abundance and important ecological role, the morays are generally difficult to study and to collect, due to their elusive nature; they are nocturnal, with cryptic behaviour. The taxonomy of the genera is still controversial, and their phylogenetic relationships have not been well established [10,11,12,13,14,15].

Cytogenetic studies can provide genetic data at the chromosome level that may assist in elucidating taxonomic issues, such as the understanding of complex or cryptic species [16,17]. According to Molina et al. [18], Actinopterygii karyotype structure shows two clear trends: clades with a karyotypic prevalence of high numbers of bi-brachial chromosomes, and clades with a karyotypic prevalence of high numbers of mono-brachial chromosomes. Anguilliformes, few species of which have been studied, show karyotype assemblages in which both trends are present. With respect to the structure of the karyotype, among the four monophyletic groups into which the Anguilliformes are subdivided, a clear differentiation is evident between the Muraenoidea and the Anguilloidea and Congroidea.

The Anguilloidea and Congroidea share the modal chromosome number 2n = 38, with a prevalence of meta-submetacentric chromosomes. While the family Anguillidae (Anguilloidea) shows a conserved karyotype structure among species [19], in the most successful family, Ophichthidae (Congroidea), marked karyotypic diversity is present, in terms of both diploid and arm numbers [20].

The Murenidae is the most species-rich and studied family among the Muraenoidea. Cytogenetically, the moray species show a quite conserved chromosome number of 42; only 4 out of 21 species are characterised by different chromosome numbers: *G. kidako* (2n = 36) [21], *G. moringa* (2n = 44) [22], *G. javanicus* (2n = 40), and *G. flavimarginatus* (2n = 36) [23]. Despite chromosome number conservation, strong cytogenetic diversity is present among morays, with karyotypes ranging from mainly bi-brachial in *G. miliaris* to all mono-brachial in *G. pictus*, and with a prevalence of karyotypes showing many subtelo-acrocentric chromosomes (see the table I in Coluccia et al. [23]).

However, cytogenetic data are still scarce for the whole Muraenidae family. Only 21 species have been studied, and these studies have been restricted mainly to heterochromatin and nucleolar organizer region localisation by silver staining (AgNORs); furthermore, the mapping of major rDNA and telomeric sequences using fluorescence in situ hybridisation (FISH) was achieved in nine species (see the Table I in Coluccia et al. [23]) [22]. Only three species (*Muraena helena*, *G. unicolor*, and *G. tile*) were analysed and compared by replication banding, and the conservation of many chromosome pairs among these three species has been reported [24,25]. Furthermore, between *M. helena* and *G. unicolor,* pericentric inversions and differences in heterochromatic amount and structure have been demonstrated by restriction endonuclease banding and FISH mapping of specific repetitive sequences [26,27,28,29].

With the aim of extending our understanding of the karyology of this fish group, mapping of the 5S ribosomal gene family using FISH was conducted on seven species: the Atlantic-Mediterranean moray *Muraena helena* L., and six species of the genus *Gymnothorax* Bloch, 1795 of different geographic origin: the fimbriated moray *G. fimbriatus* (Bennett, 1832), the yellow-edged moray *G. flavimarginatus* (Rüppell, 1830), the giant moray *G. javanicus* (Bleeker, 1859), the Indian mud moray *G. tile* (Hamilton, 1822), and the undulated moray *G. undulatus* (Lacépède, 1803), all of Indo-Pacific origin, and the Atlantic-Mediterranean brown moray *G. unicolor* (Delaroche, 1809).

The chromosomal localisation of 5S rDNA sites was compared with the major ribosomal gene sites previously localised [23,25,26,28]. In some species, the two ribosomal gene families were detected nearby positions on the same chromosome; therefore, dual colour FISH experiments using both ribosomal probes were conducted to detect possible association on the same pair. Our results revealed a variability in the number and location of 5S rDNA clusters, in some cases syntenic with 45S rDNA or associated with heterochromatic regions, and confirmed a greater conservation of the number and location of the 45S rDNA clusters among species.

## 2. Materials and Methods

Four specimens of the Indopacific *Gymnothorax javanicus* (one female, one male, and two of undetermined sex), one female of *G. flavimarginatus*, two females of *G. undulatus*, and two specimens of *G. fimbriatus* (one male and one of undetermined sex) were collected along the southern coral reef of North Male atoll in the Maldives Islands (Indian Ocean) with a fishing line while snorkelling, at a depth of 2–5 m. The sampling was performed in accordance with Maldivian environmental legislation (Research Permission OTHR/30 INDIV/2010/1163). Two specimens of the Indopacific *G. tile* (one female and one specimen of undetermined sex) were bought in an aquarium shop, coming from the swamps of the State of Pahang in Malaysia, while six specimens of the Atlantic-Mediterranean *Muraena helena* (one female, one male, and four of undetermined sex) and three of *G. unicolor* (one male and two of undetermined sex) were captured along the southwestern Sardinian coast of the Mediterranean sea, and raised in an aquarium.

The individuals were anaesthetised using ethyl 3-aminobenzoate methanesulphonate (Sigma Aldrich; Merck life Science, Milan, Italy) in seawater, and *c*. 1 mL of blood was collected from the caudal artery using a heparinised syringe. Whenever necessary, the specimens were sacrificed with an overdose of anaesthesia, without causing more than slight pain or distress. The sex was determined by macroscopic observation and histological analysis of the gonads. Metaphase chromosomes were obtained by lymphocyte culture, as described by Salvadori et al. [24].

FISH experiments were carried out using species-specific 5S rDNA probes obtained by PCR amplification from the total DNA of each moray, using the primers 5SF and 5SR as described by Pendás et al. [30], following the amplification conditions reported in the paper. The probes were biotinylated using a nick translation kit (Roche, Merck life Science, Milan, Italy) according to the manufacturer’s instructions. Hybridisation was performed according to Coluccia et al. [28]. The FISH signals were detected with extravidin-FITC conjugate (Sigma Aldrich), and chromosomes were counterstained using propidium iodide (Sigma Aldrich).

Dual colour FISH experiments were performed using, in addition to the 5S probe, a 28S rDNA probe obtained by PCR of *Anguilla anguilla* DNA, using the primers D2F and D2R described by Zardoya and Meyer [31], following the amplification conditions reported in the paper. The two probes were labelled with biotin-16-dUTP (Roche) or digoxigenin-11-dUTP (Roche), and the FISH signals were detected using avidin-rhodamine conjugate (Vector Laboratories, Burlingame, CA, USA) and anti-digoxigenin-FITC conjugate (Sigma Aldrich), respectively. Chromosomes were counterstained using 4′,6-diamidino-2-phenylindole (DAPI) (Sigma Aldrich). All slides were mounted in Vectashield anti-fade medium (Vector Laboratories, Burlingame, CA, USA).

C-banding was performed after FISH according to Cau et al. [32]. Metaphases were observed under a Zeiss Imager M1 fluorescence microscope, and images were captured with a Hamamatsu digital camera C8484 (www.hamamatsu.com), and processed using the Cromowin Plus FISH-dedicated image analysis system (TESI Imaging; Milano, Italy).

More than 30 metaphase plates were analysed for each moray species. The arm number was calculated by counting the metacentric and submetacentric chromosomes as bi-armed, and the subtelocentric and acrocentric chromosomes as uni-armed. The chromosomes were classified according to Levan et al. (1964) [33]. Idiograms of the species were constructed on the basis of the relative length and centromeric index of each chromosome pair obtained from the analysis of at least 15 Wright’s stained and C-banded karyotypes, as previously described [23,25,27,32].

## 3. Results

**Figure 1:**
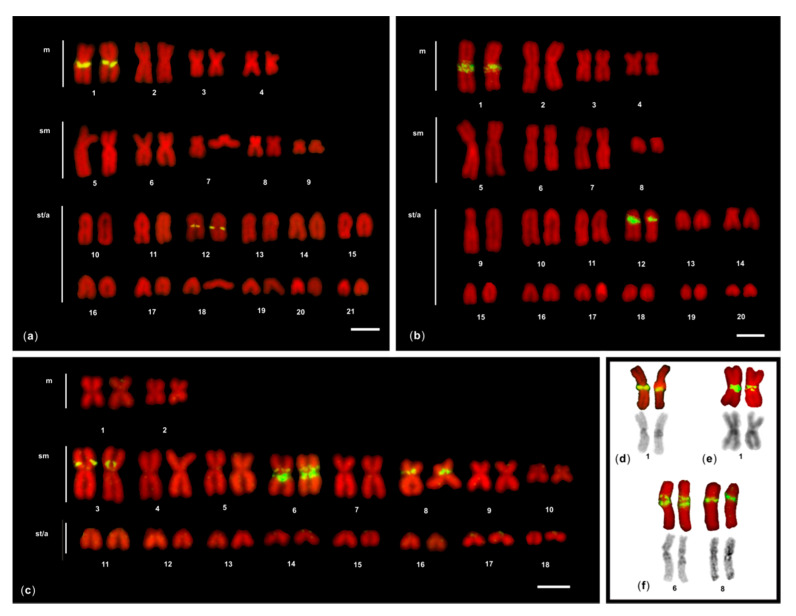
Karyotypes of (**a**) *Muraena helena*; (**b**) *Gymnothorax javanicus;* and (**c**) *G. flavimarginatus* after 5S rDNA (yellow fluorescence)FISH. The chromosomes were counterstained with propidium iodide. *m*: metacentric chromosomes, *sm*: submetacentric chromosomes, *st/a*: subtelocentric/acrocentric chromosomes. In the inset, the bi-armed 5S-bearing pairs of (**d**) *M. helena*; (**e**) *G. javanicus;* and (**f**) *G. flavimarginatus* after sequential FISH (above) and C-banding (below) showing the overlapping of 5S markings and hererochromatic C-band. The bar represents 5 μm.

**Figure 2:**
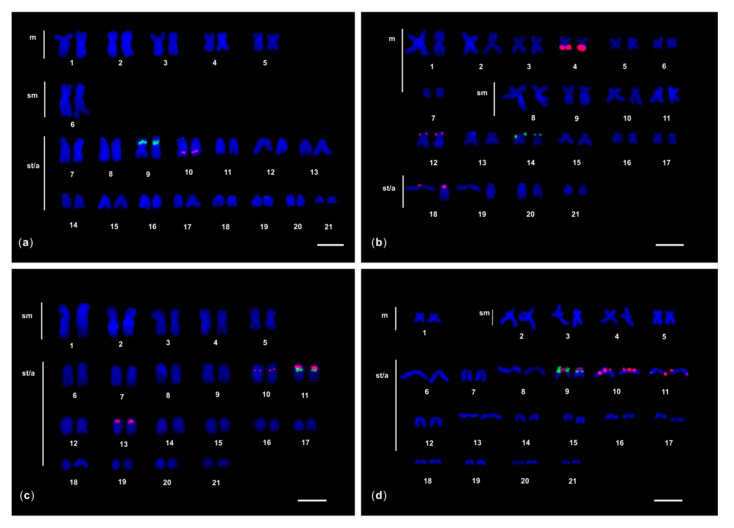
Karyotypes of (**a**) *G. unicolor*; (**b**) *G. tile;* (**c**) *G. undulatus;* and (**d**) *G. fimbriatus* after 5S (red fluorescence) and 28S (green fluorescence) rDNA dual colour FISH. The chromosomes were counterstained with DAPI. *m*: metacentric chromosomes, *sm*: submetacentric chromosomes, *st*: subtelocentric chromosomes, *a*: acrocentric chromosomes. The bar represents 5 μm.

**Figure 3:**
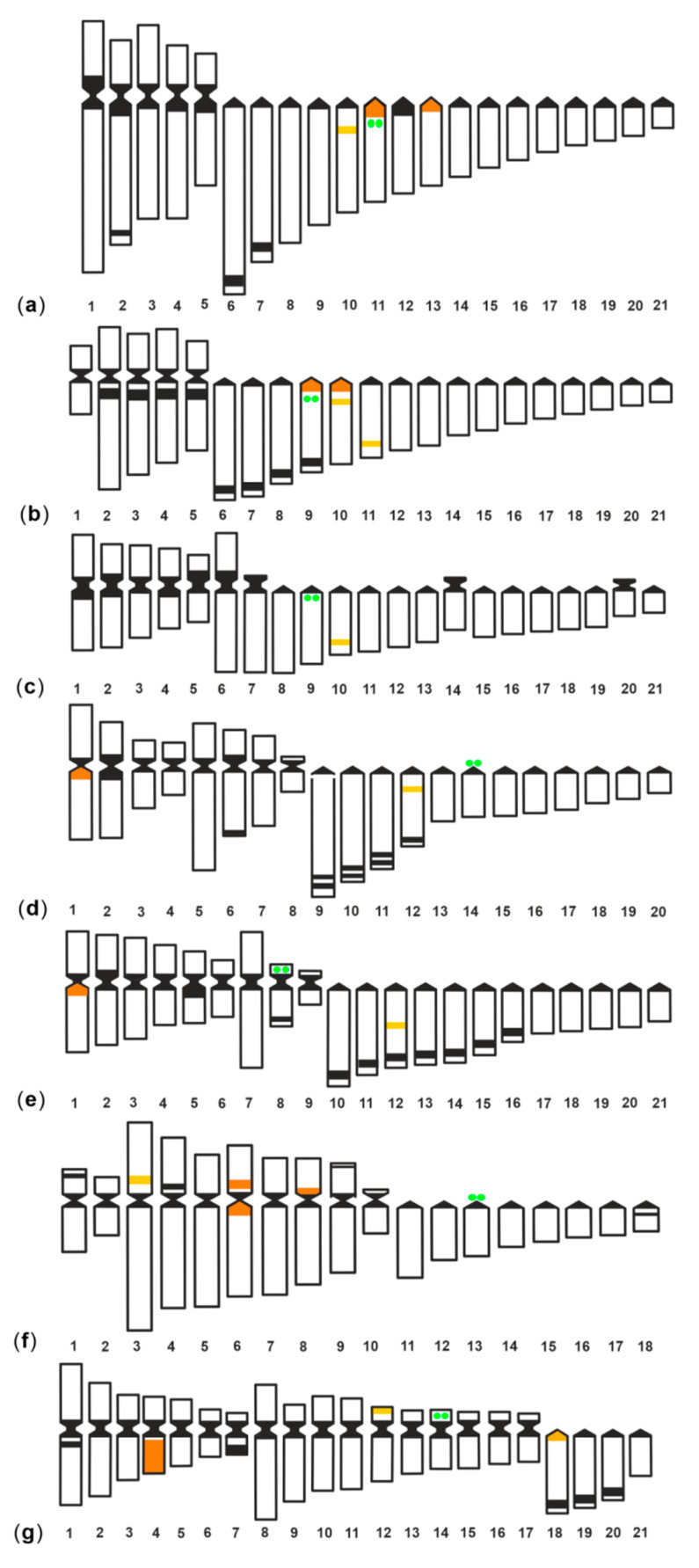
Compared idiograms of (**a**) *Gymnothorax undulatus*; (**b**) *G. fimbriatus*; (**c**) *G. unicolor*; (**d**) *G. javanicus*; (**e**) *Muraena helena*; (**f**) *G*. *flavimarginatus*; and (**g**) *G. tile* showing the C banding pattern (black regions), the 45S rDNA sites (green coloured) and the 5S rDNA sites (yellow coloured). The regions where the 5S rDNA clusters where located in heterochromatic regions are orange coloured. Chromosomes are ordered and numbered first into three groups (metacentric, submetacentric, and subtelocentric/acrocentric) and then by decreasing size within each of the three groups.

*Muraena helena*, *Gymnothorax unicolor*, *G. tile*, *G. undulatus*, and *G. fimbriatus* had the diploid number 2n = 42, while *G. javanicus* had 2n = 40, and *G. flavimarginatus* had 2n = 36 (Figure 1, Figure 2 and Figure 3) [23,25,32,34].

The karyotypes of moray species were organized into three groups: the first group included the metacentric pairs, the second the submetacentric pairs, and the third the subtelocentric and acrocentric pairs. In some cases, the numbering of the chromosomal pairs was different from that of our previous papers in which the meta- and submetacentric pairs were combined into a single group.

### 3.1. FISH of 5S rDNA

After FISH of 5S rDNA probe, one to three chromosome pairs showed fluorescent signals in the seven moray species studied (Figure 1 and Figure 2). Different chromosomal locations of 5S rDNA signals were observed: close to the centromere (proximal), interstitial, and in the terminal region (distal) of the chromosomes.

#### 3.1.1. *M. helena* and *G. javanicus*

In both species, four 5S rDNA FISH signals were detected, with a similar location: proximal in the metacentric chromosome pair 1, and interstitial in the acrocentric chromosomes 12 (Figure 1). The 5S rDNA signals located on the bi-armed chromosome pair 1 overlapped a heterochromatic C-positive region (Figure 1).

#### 3.1.2. *G. flavimarginatus*

In this moray, eight 5S rDNA FISH signals were detected, all located on three bi-armed chromosome pairs: interstitially on the *p* arm of the pair 3, proximally in the pair 8, and both interstitially on the *p* arm and proximally in the pair 6 (Figure 1). The 5S rDNA signals located on the bi-armed chromosome pairs 6 and 8 overlapped a heterochromatic C-positive region (Figure 1).

### 3.2. Dual Colour FISH of 5S rDNA and 28S rDNA

In *G. unicolor*, *G. tile*, *G. undulatus*, and *G. fimbriatus*, some of the 5S rDNA FISH signals were localised in a similar position to the previously reported 45S rDNA sites (Figure 2) [23,25,28]; therefore, dual colour FISH experiments were performed to detect a possible association.

#### 3.2.1. *G. unicolor*

In this species only two 5S rDNA hybridisation signals were detected interstitially on the acrocentric chromosome pair 10. The dual colour FISH highlighted that the 28S rDNA probe also labelled an interstitial region of the similar but distinct acrocentric chromosome pair 9 (Figure 2).

#### 3.2.2. *G. tile*

In this moray six 5S rDNA FISH signals were observed: two of them labelled most of the *q* arm of the metacentric chromosome pair 4, completely overlapping a C-positive band (datum not shown), two signals were terminally located on the *p* arm of the submetacentric chromosome pair 12, and the last two were detected proximally on the acrocentric pair 18 (Figure 2). The two 28S rDNA FISH signals were localised in a terminal position of the *p* arm of the similar but different submetacentric chromosome pair 14.

#### 3.2.3. *G. undulatus* and *G. fimbriatus*

In both species the 5S rDNA probe labelled three acrocentric chromosome pairs, one of which was also labelled by the 28S rDNA probe in a very close but more distal position (Figure 2).

In *G. undulatus*, six 5S rDNA FISH signals were localised: (*i*) interstitially in the acrocentric pair 10; (*ii*) proximally in the acrocentric pair 11, adjacent to the two 28S rDNA signals, which were distally located; and (*iii*) proximally in the acrocentric pair 13 (Figure 2). In *G. fimbriatus,* eight 5S rDNA FISH signals were localised: (*i*) two proximally in the acrocentric pair 9, adjacent to the two 28S rDNA signals, which were distally located; (*ii*) four signals were detected in the acrocentric pair 10, both in proximal and interstitial position; and (*iii*) two signals were found in interstitial position on the acrocentric pair 11 (Figure 2). In both species, all the proximal 5S rDNA signals were located in heterochromatic C-positive regions.

The chromosomal pattern of the two ribosomal gene families in the morays studied is summarized in Table 1.

## 4. Discussion

Because of their clustered organisation, the chromosomal mapping of the multigene ribosomal families—the major ribosomal DNA (45S rDNA) cluster coding for 28S, 5.8S, and 18S rRNA and located in the NOR, and the minor rDNA coding for 5S rRNA [38]—has proven to be effective in detecting micro-structural rearrangements in the karyotype of closely related species [39,40,41].

The 5S rRNA gene structure consists of a 120-bp coding sequence, conserved among vertebrates, and interspersed non transcribed spacers (NTSs) variable both in length and in sequence, making this gene family important for evolutionary studies as species-specific or population-specific markers [26,40,41]. The NTS variations are due to deletions, insertions, internal subrepeats, base substitutions, and pseudogenes; often transposable elements are also present ([42,43,44,45,46] among others).

Data reported here for Muraenidae add to the few 5S rDNA localisation available for the order Anguilliformes, where these genes have been physically mapped in only four species (see Table 1).

In the 7 Muraenidae species studied, a total of 19 5S rDNA sites were found (per haploid genome), 1 to 4 5S rDNA sites per species were present, 90% of which were in internal positions, in accordance with the most frequent situation found in vertebrates, and considered beneficial for the protection of these sequences from rearrangements [45] (Table 1). In two species, *G. fimbriatus* and *G. flavimarginatus*, one chromosome pair carries two 5S clusters.

Among the different locations, the proximal sites account for 47% and the interstitial for 42%. *G. tile* is the only species having a 5S rDNA cluster distally located, and another 5S rDNA scattered along an entire *q* arm. In all species except *G. flavimarginatus*, at least one interstitial 5S rDNA site is present in an acrocentric pair, and this is also the location of the only cluster present in *G. unicolor*; therefore, this arrangement could represent the ancestral state for the family (Figure 3).

The pattern of chromosomal distribution of the 5S rDNA is very similar between *G. javanicus* and *M. helena*, suggesting the possible homeology of the chromosome pairs 1 and 12 of both species. A similarity can also be observed between *G. undulatus* and *G. fimbriatus*, with 5S rDNA sites located in three acrocentric pairs. Additionally, in this case, the maintenance of homoeologous chromosomal segments might be hypothesised. In particular: (*i*) in the pairs 11 of *G. undulatus* and 9 of *G. fimbriatus*, 5S rDNA and 45S rDNA have a syntenic arrangement; (*ii*) in the pairs 13 of *G. undulatus* and 10 of *G. fimbriatus*, a proximal 5S rDNA site is present; furthermore, in *G. fimbriatus*, the presence of an additional 5S rDNA cluster might indicate the occurrence of a duplication; (*iii*) in the pairs 10 of *G undulatus* and 11 of *G. fimbriatus*, an interstitial 5S rDNA site is present with a different location, probably due to a small inversion.

*G. tile* and *G. flavimarginatus* show the most differentiated 5S rDNA chromosomal patterns among the moray studied; this differentiation is also observable in the karyotype structure consisting of a prevalence of bi-armed chromosomes, unlike the other species in which uni-armed chromosomes prevail (Figure 3).

In *G. flavimarginatus*, the lack of 5S rDNA clusters in acrocentric chromosomes and their presence in bi-armed chromosomes might suggest that three acrocentric pairs carrying the 5S rRNA genes may have been involved in centric fusions that gave rise to the pairs 3, 6, and 8. These data reinforce the hypothesis that Robertsonian rearrangements have occurred, leading to a karyotype of 36 chromosomes, the lowest diploid number found in morays to date [23].

The variability of rDNA sites could be due to the ability of ribosomal DNA to spread through the genome, thus generating new copies of rDNA, also in association with other repeated sequences [46]. In the morays studied, an association of 5S rDNA clusters with heterochromatic regions was observed for most of the sites located on bi-armed chromosomes, as well as for most of the proximal sites of acrocentrics (orange coloured in Figure 3), and in *G tile*, an interspersion between 5S rDNA and heterochromatin is observed along the entire *q* arm of the pair 4. As heterochromatic regions could represent recombination hotspots [46], in morays, they could have favoured the spread of associated 5S rDNA, leading to the variation in the number of clusters observed. The number and localisation of AgNORs/45S rDNA clusters are substantially conserved in the Muraenidae, as in general in the Anguilliformes. In all morays, only one major ribosomal site was reported, with the terminal location on a submetacentric/acrocentric pair as the most frequent condition; in only four species (*G. unicolor*, *G. fimbriatus, G. undulatus*, and *G. vicinus*) was the 45S site detected interstitially in an acrocentric chromosome pair [23,34,47].

Combining data on the localization of the two ribosomal gene families in Muraenidae, the 28S rDNA and one of the 5S rDNA clusters are associated in an acrocentric chromosome pair, with a highly similar contiguous position in both *G. undulatus* and *G. fimbriatus*. This syntenic condition is not common in fish, having been observed in only 20% of the species analysed [48], including *C. conger* among Anguilliformes [37]. The similar syntenic distribution of the two ribosomal families further confirms the high degree of conservation of the karyotype of the two species, and supports their close relationship, as reported in several molecular phylogenetic reconstructions of morays [5,10,49]. Unfortunately, only some of the moray eel species we analysed were included in molecular phylogenetic studies, making a deeper comparison with our cytogenetic results difficult.

## 5. Conclusions

In conclusion, the present study extended for the first time the cytogenetic knowledge of the Muraenidae family by chromosomal mapping of the 5S ribosomal gene family in seven species of different geographical origin, and two different genera. The results show variability in the number and location of 5S rDNA clusters and confirm a substantial conservation of the number and location of AgNORs/45S rDNA clusters. These data are in contrast to the general situation reported for fishes, where variability of 45S rDNA clusters and stability of 5S rDNA loci are more frequently observed [45,50,51].

## Figures and Tables

**Table 1:** Chromosomal pattern of ribosomal genes in Anguilliformes; 2n (diploid chromosome number), NF (fundamental arm number), sites (*N* number of sites), pairs (*N* number of chromosome bearing pairs), location: i (interstitial), d (distal), p (proximal), chrom: chromosome pair, a (acrocentric), *p*arm (short chromomomal arm), *q*arm (long chromosomal arm). Each species has a single locus for 45S rDNA.

Species	2n	NF	45S rDNA Location/Chrom	5S rDNA			References
				Sites (*N*)	Pairs (*N*)	Location/Chrom	
Family Muraenidae							
*Gymnothorax undulatus*	42	52	i/a11	3	3	i/a10; p/a11; p/a13	[23], present study
*G. fimbriatus*	42	52	i/a9	4	3	p/a9; p/a10; i/a10; i/a11	[23], present study
*G. unicolor*	42	54	i/a9	1	1	i/a10	[28], present study
*G. javanicus*	40	56	d/a14	2	2	p/*q*arm1; i/a12	[23], present study
*Muraena helena*	42	60	d/*p*arm8	2	2	p/*q*arm1; i/a12	[29], present study
*G. flavimarginatus*	36	56	d/a13	4	3	i/*p*arm3; p/*p*arm6; i/*p*arm6; p/*p*arm8	[23], present study
*G. tile*	42	76	d/*p*arm14	3	3	*q*arm4; d/*p*arm12; p/A18	[25], present study
Family Anguillidae							
*Anguilla anguilla*	38	60	d/*p*arm8	1	1	p/a19	[19,35]
*A. rostrata*	38	60	d/*p*arm8	1	1	p/a19	[19,36]
Family Congridae							
*Conger conger*	38	50	p/a19	1	1	p/a19	[37]
Family Ophichthidae							
*Ophisurus serpens*	38	♀ 76 ♂ 75	d/*p*arm9	♀ 4 ♂ 3	♀ 2 ♂ 1 + X	♀d/*p*arm15; d/*p*armX ♂ d/*p*arm15; d/*p*armX	[20]
